# Contact dermatitis in children

**DOI:** 10.1186/1824-7288-36-2

**Published:** 2010-01-13

**Authors:** Paolo Pigatto, Alberto Martelli, Chiara Marsili, Alessandro Fiocchi

**Affiliations:** 1 Department of Paediatrics. Fatebenefratelli-Melloni Hospitals - Milan, Italy; 2 Department of Technology for Health, Dermatological Clinic, IRCCS Galeazzi Hospital, University of Milan - Milan, Italy

## Abstract

Contact dermatitis in pediatric population is a common but (previously) under recognized disease. It is usually divided into the allergic and the irritant forms.

The diagnosis is usually obtained with the patch test technique after conducting a thorough medical history and careful physical examination but patch testing in infants may be particularly difficult, and false-positive reactions may occur.

This study also provides an overview of the most common allergens in pediatric population and discusses various therapeutic modalities.

## Introduction

Substances that are responsabile of contact dermatitis in pediatric age, can be irritant as chemical or fisical agents that causes irritant contact dermatitis (ICD) or sensitizers when causes a tissue inflation damage with allergic mechanism (allergic contact dermatitis (ACD)). Contact dermatitis grows in frequency reaching with age the prevalence as that of adult population. The lesions may be polymorphic, both in relation to the flare of the dermatitis and in relation to various responsible substances. Even the location can be initially linked to direct contact area, but thereafter may be involved different skin areas, even far from the starting site. All these factors explain why the diagnosis is not easy for the pediatrician, both for clinical investigations and for the recognition of the offending substances.

### The skin of the child and contact dermatitis

This is due to the fact that children's skin has a thinner stratum corneum than that of an adult, and the formation of the other layers of the epidermis is incomplete [1]; it is only at the age of puberty that it acquires the thickness and trophism typical of adult skin.

The absorption of substances in contact with the skin is greater in newborns and infants for three reasons:

1) their skin is thinner;

2) there is a high ratio between skin surface area and body weight;

3) the skin is covered by a sort of down that increases the absorbent surface.

The skin has many functions, the most important of which include acting as a physical and chemical barrier against external insults, and an immunological function mediated by a series of cells (keratinocytes, dendritic and endothelial cells, lymphocytes and mastocytes among others) that collaborate in orchestrating the immune response.

Contact dermatitis can be defined as an inflammatory process affecting the surface of the skin that is induced by contact with chemical, physical and/or biotic agents in the environment, and which lesions the skin, mucosae and semi-mucosae by means of allergic and irritant pathogenetic mechanisms [2].

The allergic form generally starts developing at the age of 2-3 years (and sometimes even as early as six months) because of progressive exposure to sensitising agents and the immaturity of the cell-mediated immune system during the first two years of life, whereas irritant contact dermatitis may develop from birth.

The clinical manifestations include localised pruritic eczematous reactions at the site of contact with the incriminated substance but, especially in the case of allergic forms, may also appear at a distance); the irritant forms may lead to erythemato-desquamative or bullous lesions.

The most frequently observed clinical pictures are classic (edemato-vesciculo-crusty), lichenoid and nummular.

### Irritant contact dermatitis

Irritant contact dermatitis (ICD) develops as a result of a direct insult to the stratum corneum that causes a change in pH or cellular lipids leading to cell activation and a visible inflammatory response.

Everyone exposed to the same irritating agent is potentially capable of responding in a stereotyped manner, although factors such as the presence of ongoing dermatitis (atopic dermatitis), environmental factors or physical traumas may facilitate onset.

The most common form of ICD in early infancy is nappy rash. The factors favouring its onset are the dampness and maceration of the area covered by the nappy, prolonged contact with organic liquids and the secondary development of ammoniac by fecal bacterial flora, the development of *Candida albicans *and inflammatory bacteria, the deodorants and preservatives contained in nappies, the creams and oils applied many times a day, and mechanical stimuli caused by physically cleaning and washing the newborn. The areas involved are the genitals and buttocks, with typical sparing of the skinfolds, and it may extend to the abdomen and lower limbs. The causes may be the pale blue, pink or green dyes [3] or rubbers contained in nappies, as has been demonstrated by the recently described "Lucky Luke dermatitis" due to the use of derivates of the rubber (mercaptobenzothiazole) and glues (*p*-terbutylphenolformaldehyde resin) used in the manufacture of some types of nappies [4].

Differential diagnosis needs to consider atopic dermatitis, seborrheic dermatitis, psoriasis, infections and the acrodermatitis enteropathica due to zinc deficiency.

Prevention is based on changing nappies frequently without using the clean part of the old nappy to dry the genital area, washing with non-aggressive, perfume- and preservative-free, neutral pH cleansers, and using topical emollients with a simple formulation. It may be useful to apply a barrier cream such as zinc oxide, which isolates the skin from irritants and has a soothing anti-inflammatory effect.

Therapy is based on the use of mildly antiseptic packs followed by the application of emulsions. Topical corticosteroids should be avoided, particularly those that are fluoridated, because they can give rise to granolomas and atrophy.

Other forms of ICD seen in the first years of life are perianal dermatitis due to undigested food particles that cause mechanical irritation during defecation, frictional dermatitis due to clothing (particularly in atopic subjects), forms due to over-aggressive cosmetics particularly if used on lesioned skin, perioral dermatitis, and irritant cheilitis which, in addition to occurring concomitantly with atopic dermatitis [5], can be induced by contact with a dummy, habitual lip biting, or during dentition as a result of excessive saliva production.

Children who play outdoors may develop phyodermatitis by means of a traumatic, chemical or toxic mechanism (airborne or the result of direct contact), which may also be favoured or worsened by exposure to the sun (contact phytophotodermatitis).

### Allergic contact dermatitis

The occurrence of allergic contact dermatitis (ACD) increases with age; prevalence rates of 13.3-24.5% have been reported [6], but the highest sensitisation rate has been found in children aged 0-3 years [7]. ACD used to be considered rare, but seems to have become a significant problem over the last years during which greater attention from pediatricians and dermatologists has increased the use of patch tests and consequently the number of diagnoses. Its prevalence is currently estimated to be between 14.5% and 70%, depending on whether the population was selected or not, the method used and the size of the sample, and has a tendency to increase with age.

Unlike ICD, ACD develops in predisposed subjects and involves an immunological mechanism that requires an initial sensitisation phase followed by the elicitation phase that causes the skin lesions. The sensitisation phase begins when the hapten penetrates the skin, where it is first biochemically transformed by epidermal enzymatic processes and then conjugated with a carrier protein to become immunogenic. The antigen is thus captured by antigen presenting cells (APCs), particularly Langherans cells, processed, bound to class II MHC molecules, and exposed on the cell surface. At this point, under the influx of the numerous cytokines produced by keratinocytes and APCs, the Langherans cells migrate towards the locoregional lymph nodes where specific effector and memory T lymphocytes are selected and clonally proliferated [8] before leaving the lymph node, entering the bloodstream and reaching the skin. At the end of this phase, the subject is sensitised to the hapten. The elicitation phase begins with a new contact and, once it has penetrated the skin, the offending substance undergoes chemical changes, and is then recognised and processed by Langherans cells. At this point, the specific T lymphocytes are recalled at skin level and, together with keratinocytes, release numerous cytokines that amplify the inflammatory response and give rise to skin damage cutaneo.

The legs and feet, hands and face are the sites most frequently affected by pediatric ACD, which is mainly caused by metals, footwear, topical medications and cosmetics. The importance of the different substances depends on the age of the subject at the time of onset, changes in lifestyle, and contact with new haptens.

Cosmetics are the main cause of ACD in early infancy because mothers are increasingly using herbal or other products containing active ingredients that are not classified as medicinal specialties. Irritant forms are more frequent than allergic forms, and are due to the application of topical agents that are too aggressive, particularly in the case of children with atopic dermatitis. Preservatives such as the parabenes, Kathon CG and Euxil 400 can cause sensitisation, especially when applied to lesioned skin or if they are used in products designed to be left in contact with the skin rather than rinsed off, and the lanolin alcohols [9,10] used because of their emulsifying and emollient propertiesi. Even so-called "natural" products such as propolis [11] or tea tree oil [12] can cause ACD.

Once again in early infancy, but also later, an important role is played by topical medicaments such as antibiotics [13] and topical cortisones [14] (also see below), and antiseptics and disinfectants including mercurochrome [15], thimerosal [16] and neomycin [17], which are very important because of their widespread use and are often found in vaccine formulations. Although very frequent, sensitivity to thimerosal is not very significant.

Contact with metals increases exponentially during school age and adolescence. Those responsible for the highest incidence of ACD during childhood are nickel, chrome [18] and cobalt, which can also induce co-sensitisation to other metals [19]. Nickel sulfate is the most frequently involved allergen as it is contained in bijoux jewellery, the metal accessories of clothing, watch bracelets, spectacle frames and orthodontic appliances. Ear piercing is the main cause of nickel sulfate sensitisation, and the risk increases with the number of piercings [20].

Additional and often unknown sources of nickel exposure are once again cosmetics and cleansing agents, which is why the use of products containing little or no nickel is recommended for children. The make-up used by children to emulate their mothers or for purposes of dressing up are often contaminated by nickel, and particularly the use of mascara, eye-liners, eye pencils and eye shadow can cause dermatitis of the eyelids. It is strongly recommended not to use products sold by nesagents or toyshops because they not suitably controlled.

Girls are also frequently sensitised to the perfumes contained in numerous products (often including those that are claimed to be "fragrance-free") in the form of essential oils or perfumed preservatives such as benzyl alcohol. The corresponding hapten positivities are "fragrance mix" and balsam of Peru [21].

As chrome (potassium dichromate) is one of the ingredients used in the tanning of leather, wearing leather shoes is the main cause of sensitisation, particularly in the summer when shoes may be worn without socks. Over the last few years, the advent of shoes prevalently made of rubber has led to a clear increase in the incidence of sensitisation to vulcanisation accelerators, rubber antioxidants (the thiurams and *p*-phenylenediamine) and glues such as resins (*p*-ter-phenolformaldehyde resin).

Orthodontic appliances made of stainless steel, chrome-cobalt and nickel-titanium [22] can cause perioral dermatitis (angular or other forms of cheilitis) and lesions of the oral mucosa. The most frequently incriminated heptens are nickel, chrome, cobalt and titanium. Such clinical pictures are rare in children (who, unlike adults, wear the appliances for just a few years) because the skin manifestations are due to the amount of metal ions released by the appliance, which increases with time and the wear of the materials.

It is also known that the inclusion of metal alloys in orthopedic prostheses can have adverse effects on sensitised subjects, although these are less frequently encountered in children. Nevertheless, caution is required when using endoprostheses in those who are known to be sensitive to metals.

In adolescence, the substances used in hair dyes (such as *p*-phenylenediamine) can cause ACD of the scalp, face and eyelids.

Over the last few years, there has been an increase in the prevalence of both ICDs and ACDs due to textiles, especially clothing. The ICDs arise from the excessive heat that may develop under certain conditions and the perspiration that collects in skinfolds (which may also be irritated by rubbing) as it favours the release of substances by the material and thus induces sensitisation. The most widely known are dyes and fixing resins, but the glues, and rubber and metal accessories of clothing can also lead to sensitisation if they remain in contact with the skin. However, given the enormous variety of fabrics and clothing on the market (particularly children's clothes), this type of ACD is clinically highly polymorphic.

It is also worth noting that sites that are not normally in contact with the fabrics, such as the hands, may be an indicator of the presence of ACD [23].

Once the hapten to which a child has been sensitised has been identified, it is possible to treat the ongoing lesions, but it is also essential to avoid any future contact with the causative substance. This is easy in some cases, but may be difficult in the case of ubiquitous haptens (e.g. nickel), or impossible if the hapten is unknown.

When giving the results of patch tests, physicians generally also offer useful information about the types of products containing the responsible hapten so that they can be avoided. Nevertheless, it is necessary to identify the ingredients whenever using cosmetics, which are defined as "the non-medicinal substances and preparations designed to be applied to the outer surfaces of the human body (epidermis, the hair and piliferous system, nails, lips and external genital orgnas) or to the teeth or the mucosa of the mouth for the exclusive or prevalent purpose of cleaning them, perfuming them, changing their appearance, correcting body odours, protecting them or maintaining them in good condition".

In Italy, the production and sale of cosmetics is governed by Law No. 713 of 11 October 1986, and Italian legislation has also assimilated the European Community Directive 76/768/CEE, which was issued to standardise the situation throughout Europe. In particular, Law No. 713/86 concerns the composition of cosmetic products, their presentation (by which is meant their labelling, packaging and every other form of external representation), and the requirements it is necessary to fulfil before producing, selling or importing them. Their composition is a fundamentally important aspect that is the subject of continuous study at EU level and is disciplined with particular care. Directive 76/768/CEE included lists of substances that were prohibited or had to be used within certain limits, and these lists are continuously updated on the basis of the recommendations made by the appropriate technical committees; Law No. 713/86 includes the same lists, which are therefore constantly updated on the basis of directives issued by the European Commission. A cosmetic product whose composition does not respect these indications is considered unlawful, and anyone producing or selling is subject to sanctions.

Medicinal products are defined as any substance or combination of substances presented as having curative or prophylactic properties relating to human diseases, and any substance or combination of substances that can be used on or administered humans with the aim of retoring, correcting or modifying physiological functions by exercising a pharmacological, immunological or metabolic action, or for the purpose of establishing a medical diagnosis. The labelling of every medicinal product of industrial origin is subject to the regulations of the Italian Medicines Agency (AIFA) or the European Medicines Agency (EMEA). The approach to so-called "natural" herbal or homeopathic products is more complex as they are not classified as medicinal products and often consist of mixtures of substances whose composition is unknown or only partially known.

### Contact dermatitis and atopic dermatitis

Children with atopic dermatitis (AD) are very often affected by contact dermatitis [24,25].

The irritant forms are the most frequent as the skin of atopic subjects is highly sensitive to external aggression due to cosmetics, cleansers and tensioactive agents, or fabrics such as wool or synthetic fibres.

However, the relationships between atopic dermatitis and ACD are more complex and still a subject of debate. The results of studies of the prevalence of ACD in DA are conflicting but, from a pathogenetic point of view, it can be said that sensitisation is more likely in periods of disease quiescence rather than exacerbation because of the shift towards a Th1-mediated reaction

It is possible that the drugs used to treat AD worsen the disease by inducing sensitisation. The topical antibiotics used in the case of the bacterial supra-infection of AD lesions may cause the onset of ACD at the level of the ano-perineal area, the eyelids, the auricles and the legs. Comparisons of topical antibiotics have shown that the phenomena related ACD are cross-sensitisation and the possibility of systemic reactions when the active ingredient is administered systemically. Topical antivirals, antimycotics, NSAIDs and antihistamines may also cause ACD; the last two in particular may cause photodermatitis.

Topical cortisones can be sensitising and, if they are, the patch test reactions are delayed and the readings are made seven days after patch removal. Even the emollients that are strongly recommended in the attack and maintenance therapy of AD can cause ACD because they contain albeit minimum amounts of tensioactive agents and preservatives. A study by Mailhol *et al*. [26] found that the cause of the onset of medicinal-induced ACD in subjects with AD was (in order of frequency) emollients, antiseptics and cortisones, and that the related rik factors were the severe disease, an early onset (at an age of <6 months), and the presence of IgE-mediated sensitisation.

In the case of AD lesions localised to typical sites (the face and convex surfaces in early infancy; the flexor surfaces, eyelids and hands in school-age children and adults) that are resistant to or worsened by topical treatments, patch tests can be useful, particularly the cortisone, cosmetic and antibiotic series, or tests of the products used by the child.

In all other cases of AD, patch tests are indicated if the subject has a history of the onset of lesions in a body region that is not characteristic of AD but is the site of contact with various substances (e.g. metals, footwear, textiles, perfumes).

Separate consideration should be given to the atopy patch tests, which include aero- and tropho-allergens capable of inducing a type I IgE-mediated hypersensitivity reaction, and a delayed reaction in skin areas tested in order to evaluate the role of allergens in maintaining or worsening the AD in the absence of any other known cause.

### Diagnosis

The essential procedure in the case of a clinical suspicion of contact dermatitis is to apply patch tests or epicutaneous tests.

Patch tests are recommended whenever there is a clinical or anamnestic suspicion of contact dermatitis or when a child not only fails to benefit from the recommended treatment for a dermatological disease such as AD and actually experiences worsening symptoms. Studies have shown that the sites most frequently affected by ACD for which the results of patch tests are relevant are the eyelids, genitals, hands and feet [27].

Patch tests involve a battery of appropriately formulated low-molecular-weight substances diluted in a solid or aqueous vehicle and contained in small reservoirs on a support that is held in place by an occlusive plaster in order to ensure contact with the skin for 48 hours. The aim is to reproduce a cell-mediated reaction (type IV of Gell and Combs) at the contact site. The results are interpreted when the patches are removed and after a further 48 hours.

Sometimes, when diagnosing a case of dermatitis induced by textile dyes, the reaction may be particularly delayed [28].

The patches are generally applied either side of the spinal column, avoiding the scapular protuberances and any areas of lesioned skin (acne, scars, etc). If it is not possible to use the back, they can be put on the side of the arm (deltoid region), the front of the thigh, or the abdomen. The patches must not be removed or moved by the patient, who has to avoid wetting the area or undertaking physical exercise throughout the duration of the test.

The reactions are assessed on the basis of visual inspection, digital palpation and subjective symptoms (pruritus, burning), and attributed a score:

0: Negative reaction

+/- or ?: Doubtful reaction: minimal erythema

+: Weakly positive: erythema and edema

++: Clearly positive: erythema-edema-vesciculation

+++: Highly positive: erythema-edema-bullae

IR: Irritant reaction

In pediatrics, 15-52% of patch tests prove to be positive.

The tests are sufficiently standardised for adults but not for children. Some authors suggest using lower hapten concentrations in order to avoid false positive reactions, but the majority now agree on using the same concentrations as those used for adults and being more cautious in interpreting the responses. Thresholds for the sensitisation induced by dispersed textile dyes have also been studied [29].

There are now many hapten batteries available: the standard series, the supplementary series, and the specific series to be used as necessary.

Different substances may be used in patch testing for different children ages (table 1).

**Table 1:** The series used in children.

Patch test series	Indications
Pediatric	0-14 years

Cosmetic	≥6 months

Eyelid	Adolescence

Fragrances	Adolescence

Footwear	≥6 years

Metals and orthopedic	≥6 years

Corticosteroid	AD or other dermatological diseases

Antibiotic	AD or other dermatological diseases

Detergent/disinfectant	AD or other dermatological diseases

Photopatch test	≥14 years

Textiles	≥2 years

Hairdressing	≥14 years

### Therapy

The treatment of contact dermatitis consists of using of topical or systemic steroids depending on severity but, above all, avoiding contact with the offending substances and those that may be involved in allergic cross-reactivity [30]. However, avoidance is not always possible: for example, nickel is contained in many foodstuffs [31] and found as an impurity in metallic and other objects, and cosmetics often contain traces of fragrances that are not included in the published product specifications.

In the acute phase, mildly antiseptic solutions and emollient creams are recommended, and low-strength corticosteroids can be used locally for a brief period.

In the case of chronic forms, the use of more potent corticosteroids is useful, as are moisturising agents in a greasy vehicle for thicker skins.

## Conclusion

Contact dermatitis is underestimated in children because the diagnostic procedure requires specific skills in of some diagnostic techniques, such as patch tests, not always easy to achieve. The haptens that most frequently cause of pediatric contact dermatitis are nickel, cobalt and other some metals such as chromium and fragrances. The collaboration between pediatrician and dermatologist is strongly needed not only for diagnosis but also for monitoring new enviromental substances that, with increasing frequency, are used in the childhood. Only through a careful recognition of substances it is possible a correct CD diagnosis and also a prompt removal of responsible aptens.

## Competing interests

The authors declare that they have no competing interests.

## Authors' contributions

All authors read and approved the final manuscript.
